# Effectiveness of Tocilizumab in the Treatment of a Recent Kidney Transplant Recipient With COVID-19

**DOI:** 10.7759/cureus.53163

**Published:** 2024-01-29

**Authors:** Elisabed Chikobava, Lasha Chkhikvadze, Keti Menabde, Giorgi Pkhakadze, Irma Tchokhonelidze

**Affiliations:** 1 Faculty of Medicine, Tbilisi State Medical University, Tbilisi, GEO; 2 Department of Nephrology, High Technology Medical Center Hospital, Tbilisi, GEO

**Keywords:** covid-19, interleukin 6, immunosuppression, kidney transplantation, tocilizumab

## Abstract

The coronavirus disease 2019 (COVID)-19 pandemic significantly affected transplantation care strategies due to the heightened vulnerability of transplant recipients to severe illness. We present a unique case of a 31-year-old female with COVID-19 pneumonia following a recent kidney transplant managed with immunosuppressant reduction and tocilizumab therapy. The patient underwent live donor kidney transplantation and was considered a low immunologic risk recipient. Following surgery, she presented with fever, headache, and fatigue, and subsequent testing confirmed active COVID-19 infection. Imaging revealed characteristic pneumonia features. Standard approaches, including immunosuppressant reduction and antibiotic therapy, initially failed to halt clinical deterioration. Progressive radiological findings and increasing inflammatory markers raised concerns of impending graft failure and cytokine storm. Considering the severity of the condition, tocilizumab, an interleukin-6 (IL-6) receptor antagonist, was administered alongside continued supportive care and adjusted immunosuppression. Within a day post-tocilizumab infusion, the patient showed significant improvement in clinical parameters, with resolution of respiratory distress and systemic symptoms. Laboratory markers gradually normalized, and subsequent lung imaging showed improvement. The patient was discharged with follow-up recommendations. Managing COVID-19 in postoperative transplant patients requires nuanced approaches due to immunosuppression-related complexities. Despite limited guidance, our case highlights the successful use of tocilizumab in treating COVID-19 pneumonia shortly after transplantation, showcasing its potential effectiveness and safety in this context. Reporting such experiences is crucial for refining management strategies for immunocompromised transplant recipients facing COVID-19 complications.

## Introduction

Transplant recipients, often immunosuppressed to prevent organ rejection, are at a higher risk of severe illness if they contract any type of infection [1]. For this reason, the coronavirus disease 2019 (COVID-19) pandemic drastically impacted the transplantation planning strategy and postoperative care of recipients. Some general guidelines existed for transplant patients to manage various viral infections [2]; however, the unexpected appearance of COVID-19 required incorporating novel approaches into standard care. We had to face such a case during the pandemic in Georgia. Herein, we report a case of COVID-19 pneumonia following kidney transplantation, which was successfully managed with immunosuppressant reduction coupled with tocilizumab therapy. We assume that our intervention protocol may serve as a template for the treatment of other such patients.

This case was previously presented as an abstract at the 21st Century Medicine - International Medical Congress for Students and Young Doctors on June 3, 2023.

## Case presentation

**Figure 1:**
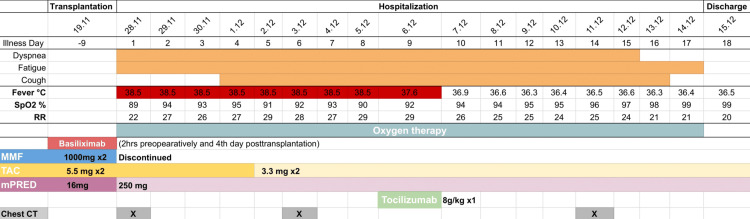
Symptoms and treatment, including immunosuppressive adjustment according to the day of illness and day of hospitalization MMF: Mycophenolate mofetil; TAC: Tacrolimus; mPRED: Methylprednisolone

The patient was a 31-year-old female who underwent live donor kidney transplantation (LDKT) on November 19, 2020, with her father being a donor. The patient was assessed as a low immunologic risk recipient (white race, less than one HLA mismatch, panel reactive antibody 0, first transplant, blood group compatibility, recipient average age 31, no presence of donor-specific antibody, and no delayed onset of graft function). She was diagnosed with focal segmental glomerulosclerosis (FSGS) in 2012 and end-stage renal disease (ESRD) in 2019. She had been on hemodialysis for the last 10 months. Induction agent basiliximab (20 mg IV) was given two hours preoperatively and on day four postoperatively following local guidelines. A triple immunosuppression regimen consisting of methylprednisolone (16 mg), mycophenolate mofetil (1000 mg BID), and tacrolimus (5.5 mg BID) was initiated. Since the patient was at a high risk of superinfection during hospitalization, she was given ceftriaxone (1 g), trimethoprim-sulfamethoxazole (TMP-SMX) (960 mg, 1⁄2 tablet), nadroparin (0.4 mL), nystatin (0.5 g TID), valganciclovir (450 mg), and fluconazole (150 mg) with the aim of prophylaxis. On postoperative day eight, she developed a fever, headache, and fatigue (Figure 1). On evaluation, the vital signs were blood pressure (BP) 120/70 mm/Hg, heart rate (HR) 105, respiratory rate (RR) 22, oxygen saturation (SpO_2_) 93% on ambient air, and temperature (T) 38.5C°. Considering her symptoms and local epidemiological situation, a rapid antigen test and polymerase chain reaction (PCR) for severe acute respiratory syndrome coronavirus 2 (SARS‑CoV‑2) were performed, and an active COVID-19 infection was confirmed. She was admitted to hospital-based isolation as the local government protocol required.

The patient’s laboratory and radiological results were as follows: WBC counts at the baseline values; the neutrophil percentage was higher (76%); and C-reactive protein (CRP) was slightly elevated (5.32 mg/l). Chest computed tomography (CT) scan showed average intensity, subpleural, segmental, nodular-infiltrating, and ground glass-type infiltrative changes in both lung parenchyma. The right lung showed damage in the upper lobe (1 point), middle lobe (1 point), and lower lobe (1 point) while the left lung exhibited damage in the upper lobe (1 point), middle lobe (1 point), and lower lobe (2 points). Minimal pleural effusion was observed bilaterally, and a small fluid collection was noted near the transplanted kidney, adjacent to the right iliac wing, and close to the lesser pelvis (Coronavirus disease 2019 (COVID-19) Reporting and Data System 6 (CORADS-6) (CT-SS-7).

These abnormalities suggested the presence of COVID-19 pneumonia. Therefore, the decision was made to withhold MMF completely, decrease the dose of tacrolimus (3 mg BID) to keep its serum concentration in the range of 7-10 ng/mL, and increase the amount of methylprednisolone (250 mg IV). A blood culture was sent and meropenem (1 g IV) plus doxycycline (100 mg PO) were started. The patient was given an antipyretic paracetamol, oxygen therapy 7-8 mL/min, and fluid resuscitation with lactated Ringer (200 mL/h) as a supportive treatment.

**Figure 2:**
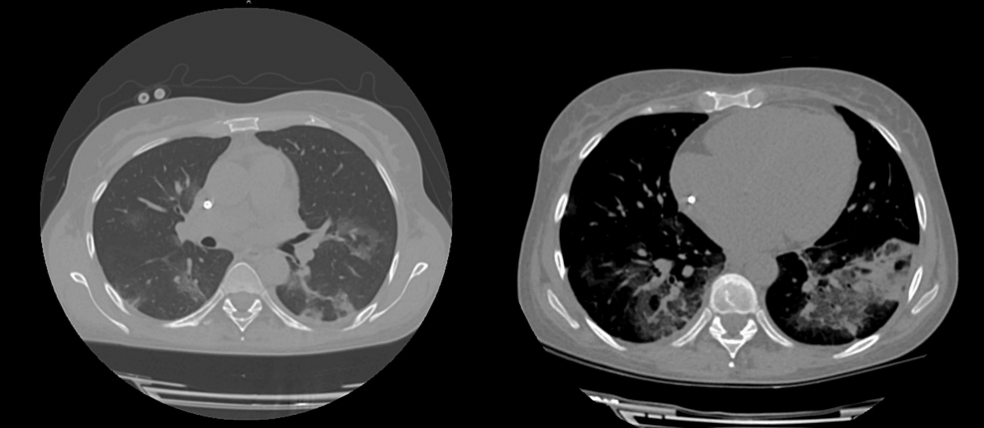
Chest CT taken on the sixth day of hospitalization

Despite taking the measures instituted by the local protocols, the clinical deterioration was evidenced by the worsening symptoms, vital signs, and dynamic CT findings over the next week. Inflammatory markers (CRP, ferritin, interleukin-6 (IL-6)) were gradually increasing (Table 1), hence respiratory distress was anticipated. The second CT was taken on the sixth day of hospitalization, and it revealed total bilateral ground glass opacities (CORADS-6) (CT-SS-22) (Figure 2).

**Table 1:** Clinical laboratory results Note: Values in bold were either above normal or below normal. RBC, red blood cell; HGB, hemoglobin; WBC, white blood cell; NEUT, neutrophil; LYMPH, lymphocyte; MONO, monocyte; PLT, platelet; sCr, serum creatinine; CRP, C-reactive protein; ESR, erythrocyte sedimentation rate; IL, interleukin

Measure	Unit	Reference range	28.11.20	30.11.20	03.12.20	05.12.20	06.12.20	07.12.20	11.12.20	15.12.20
RBC	10^12/L	3.8 - 5.8	2.4	2.7	2.6	2.4	2.7	3	3	3.2
HGB	g/dL	12-16	8.7	8.6	9.1	8.5	8.6	9.8	9.7	10.3
WBC	10^9/L	3.5 - 10	4.2	7.4	8.9	7.4	6.3	7.4	8.5	9.3
NEUT %	%	50-60	76	73.3	86.7	88.1	87.8	88.5	86.3	75.5
NEUT #	10^9/L	1.5 - 7.5	3.1	5.4	7.8	6.5	5.5	6.5	7.4	7.1
LYMPH %	%	18 - 45	10.4	15.5	9.7	10	10.4	9	11.4	18.3
LYMPH #	10^9/L	1.32 - 3.57	0.4	1.2	0.9	0.7	0.7	0.7	7.4	1.7
MONO %	%	4-10	12.8	11.2	3.2	1.5	1.8	1.7	1.8	4.2
MONO #	10^9/L	0.25 - 0.82	0.5	0.8	0.3	0.1	0.1	0.1	0.1	0.4
PLT	10^9/L	150-400	185	171	184	150	131	195.4	182	205
sCr	μmol/L	45-84	84	90	105	100	70	97	95	71
CRP	mg/L	<5	5.3	6.8	26.5	49.9	87.1	79.1	60.1	22.5
ESR	mm/h	2-27	20	20	30	22	40	45	45	20
IL-6	pg/mL	<7	5.2	10	4.4	102.7	104.7	2077	529.7	721.3

On day eight of hospitalization, the patient developed decreased urine output with increasing creatinine levels, and concern arose regarding potential transplant failure. In addition, by the end of the week, serum IL-6 levels had a hundredfold increase (Table 1), suggesting the possibility of an impending cytokine storm. Consequently, a decision was made to administer a single IV infusion of anti-IL-6 receptor monoclonal antibody, tocilizumab, 162 mg (8 mg/kg). The day after the tocilizumab infusion, significant improvement in the clinical picture was seen: T-36.5C° and increased urine output >3000 mL/24h. Meanwhile, immunosuppressive, antibacterial, antiviral, antimycotic, supportive, and oxygen therapies were continued.

Two weeks after hospitalization, the patient's weakness and dyspnea resolved, and there was an improvement in her vital signs: BP 130/70 mmHg, HR 76, RR 15, SpO2 97% (on ambient air), and T 36.2C°, therefore oxygen therapy was discontinued. The third chest CT showed improvement and decreased infiltrates bilaterally (CORADS-6) (CT-SS-9). Urine output was adequately proportional to fluid intake and serum Cr was normal (Table 2). The patient was discharged from the hospital and stayed under the nephrologist and transplantologist’s supervision. She was recommended to recheck the following: COVID-19 status with a PCR test in two weeks, non-contrast chest CT in one month, and laboratory tests (urinalysis, CBC, coagulation panel, Cr, CRP, IL-6, and serum tacrolimus concentration) in one month.

**Table 2:** Urinalysis results

Measure	Unit	Reference range	28.11.20	01.12.20	05.12.20	07.12.20
Color			Yellow	Yellow	Yellow	Yellow
Turbidity			Cloudy	Cloudy	Slightly cloudy	Cloudy
Specific gravity		1.015 - 1.025	1.025	1.02	1.025	1.03
pH		5 - 6.5	5	5	5	5
Leukocytes	Leu/μL	(<10) negative	25	neg	neg	neg
Nitrite		negative	neg	neg	neg	neg
Protein	g/L	(<1) negative	0.15	neg	0.15	0.3
Glucose	mmol/L	(<0.84) norm	norm	norm	norm	norm
Ketones	mmol/L	(<0.5) negative	neg	neg	neg	neg
Urobilinogen	μmol/L	(<17) norm	norm	norm	norm	norm
Bilirubin	μmol/L	(<3.4) negative	neg	neg	neg	neg
Erythrocytes	Ery/μL	(<5) negative	300	300	neg	300

## Discussion

The world has been dealing with the SARS-CoV-2 infectious pandemic from 2019 onward. For controlling the spread of disease and preventing its appearance, a significant amount of knowledge and expertise have been gathered over the years. COVID-19 is a health disaster, especially for immunosuppressed individuals, such as kidney transplant recipients who have undergone surgery within three months. Patients in the early posttransplant period are more prone to experience more severe clinical symptoms of the COVID-19 infection due to immunosuppression, as well as super-added and co-infections caused by other pathogens. Thus, cautious planning is necessary for COVID-19 management in this population. Regarding the treatment regimen, it is assumed that the distinctiveness of transplant patients may be related to the necessity to take immunosuppressive medications into account for preserving graft function. Reducing or even temporarily discontinuing immunosuppressive drugs is a standard approach to managing pneumonia caused by viral infection after kidney transplantation and gives patients the potential to gain immunity against infection [2].

It was a big challenge for us to handle this case because there was not enough information published about comparable cases at the time we encountered it. Initially, the patient was managed by the already existing guidelines and clinical experiences. MMF was discontinued, subsequently, we reduced the amount of tacrolimus until the desired concentration was achieved. In addition, the dose of methylprednisolone was increased to prevent acute graft rejection, decrease respiratory distress, and treat systemic symptoms such as fever and fatigue [3]. The conventional approach was insufficient, the clinical picture was deteriorating along with declining laboratory and radiologic evidence of lung and kidney functions. Neither of the two main challenges could be addressed in managing infection among early transplant patients - pneumonia failed to resolve and the risk of acute graft rejection was steadily rising. Furthermore, increasing IL-6 levels anticipated facing the cytokine storm, a severe immune response that can lead to organ damage and death. Considering the severity of the condition, it was assumed that giving tocilizumab would be reasonable.

Tocilizumab (TCZ) is a recombinant humanized monoclonal antibody of the immunoglobulin G1 (IgG1) subclass directed against the soluble and membrane-bound interleukin 6 receptor (IL-6R). It is usually indicated for chronic conditions (rheumatoid arthritis, juvenile idiopathic arthritis, and giant cell arteritis) [4]. Dysregulated IL-6 synthesis plays a key role in this cytokine storm, similar to what happens in autoimmune diseases and malignancy. The cytokine storm in COVID-19 is thought to be triggered by the virus itself and the host's immune response to the virus. Thus, targeting IL-6 can be considered a potential therapeutic approach for severe and critical COVID-19. At the time we encountered the case, the use of tocilizumab for such acute cases was controversial and information about effectiveness and side effects was limited.

After the administration of tocilizumab, significant improvement in the clinical picture was seen: the vital signs normalized and ESR and CRP slightly decreased, but there was a 20-fold increase in IL-6. Given that tocilizumab is a competitive inhibitor of IL-6R, it is expected that higher levels of IL-6 in the bloodstream after administration are due to the drug inhibiting IL-6R-mediated clearance as well as the disease's ongoing production of IL-6 [5]. According to a study done in Italy, after two doses of tocilizumab, 77% of patients showed improvement in respiratory distress. However, in another Chinese experience with 21 patients, including two who were critically ill, the authors reported significant clinical improvement in all the patients within five days after administration of just one dose of tocilizumab [6,7]. Therefore, as rapid clinical improvement was seen in our case, the second dose of tocilizumab was not given. After day three of tocilizumab administration, all the inflammatory markers started to gradually decrease and normalized by the time of discharge.

## Conclusions

We described the clinical characteristics and therapeutic course of the first case of COVID-19 pneumonia in a renal transplant recipient in Georgia, successfully managed with tocilizumab. Even though there are multiple cases reported and randomized control trials conducted about COVID-19 pneumonia in patients on immunosuppression after kidney transplantation, our case is exceptional due to the remarkably short interval between the surgery and contracting the virus, adding postoperative state as another risk factor for the severe clinical course of infection. Despite the co-existence of numerous risks predicting unfavorable outcomes, the management discussed above turned out to be effective, thus providing a reference case for treating such patients. We believe it is of paramount importance to report such an experience to optimize the management guidelines for immunocompromised transplant recipients with COVID-19.
